# 

**Published:** 1880-11

**Authors:** 


HERMAN MYNTER, M. D.

### CONTENTS:

PAGE.

ORIGINAL COMMUNICATIONS:

The Treatment of Nephritic Colic. By Charles G. Stockton, M. D., . . . . . 145
Easy Methods of Detecting Blood-stains. By Prof. D. S. Kellicott, . . . . . 150

CLINICAL REPORTS:

Typhoid Fever, complicated with Epistaxis and Tetanus. Reported by Joseph Fowler, M. D., . . . . . . . 155
Excision of Tumors of the Breast. By Chas. C. F. Gay, M. D., . . . . . . 159

TRANSLATIONS:

Are Measles more Dangerous in Grown-up Persons than in Children? From the Danish by Herman Mynter, M. D., . 163
The Movable Kidney. From the Danish by Herman Mynter, M. D., . . . . . 164

SELECTIONS:

How Arsenic Poisons, . . . . . . 165
Action of Iodide of Potassium upon the Elimination of Lead, . . . . . 167
The Therapeutical Value of Nitro-Glycerine, 168
Intra-Uterine Medication, . . . . . 170
Accidents that have been observed to follow Thoracentesis by Aspiration practiced for the Removal of Pleuritic Effusion, . 173
Misuse of Iron Preparations, . . . . . 174
On Glycerine in Flatulence, Acidity, and Pyrosis. By Sidney Ringer, M. D. and William Murrell, M. D., . . . . 176

SOCIETY REPORTS:

Buffalo Medical and Surgical Association, . 178

EDITORIAL:

Buffalo State Asylum for the Insane, . . 181
Buffalo Medical College, . . . . . . 182
Prof. Miner's Condition, . . . . . 182
List of Physicians Registered in the Clerk's Office of the County of Erie, . . . 183

REVIEWS, . . . . . . . . 187

